# Reannotation of Public Transcriptomic Data Identifies Candidate lncRNAs and Putative Regulatory Networks in Rhabdomyosarcoma

**DOI:** 10.3390/biomedicines14071648

**Published:** 2026-07-22

**Authors:** Jessica Zablocki da Luz, Leonardo Vinícius Barbosa, Thiago Rodrigues dos Santos, Aliciane de Almeida Roque, Camila Confortin, Amanda Beatriz Soares Fulan, Lúcia de Noronha, Deisy Morselli Gysi, Cleber Machado-Souza

**Affiliations:** 1 Faculdades Pequeno Príncipe, Av. Iguaçu, 333, Rebouças, Curitiba 80230-020, PR, Brazil; 2 Instituto de Pesquisa Pelé Pequeno Príncipe, Av. Silva Jardim, 1632, Água Verde, Curitiba 80250-060, PR, Brazil; 3 Departamento de Biologia Celular e Molecular, Universidade Federal do Paraná, Avenida Coronel Francisco H. dos Santos, 100, Jardim das Américas, Curitiba 81531-980, PR, Brazil; 4 Departamento de Biociências, Universidade Federal do Paraná, Rua Pioneiro, 2153, Palotina 85953-128, PR, Brazil; 5 Instituto de Pesquisa Carlos Chagas, Rua Prof. Algacyr Munhoz Mader, 3775, Curitiba 81350-010, PR, Brazil; 6 Departamento de Biologia Celular e Molecular e Patologia Experimental, Pontifícia Universidade Católica do Paraná, Curitiba 80215-901, PR, Brazil; 7 Departamento de Estatística, Universidade Federal do Paraná, Avenida Coronel Francisco H. dos Santos, 100, Jardim das Américas, Curitiba 81531-980, PR, Brazil

**Keywords:** rhabdomyosarcoma, lncRNA, ceRNA network, bioinformatics

## Abstract

**Background/Objectives**: Rhabdomyosarcoma (RMS), the most common pediatric soft tissue sarcoma, includes two main subtypes, embryonal (eRMS) and alveolar (aRMS), each with distinct molecular and clinical characteristics. Although cellular processes underlying RMS and differences between PAX3-FOXO1 fusion-positive and fusion-negative tumors are well known, the contribution of long noncoding RNAs (lncRNAs) remains poorly understood. **Methods**: Here, we reannotated publicly available microarray datasets to comprehensively profile lncRNA expression and reconstruct lncRNA–miRNA–mRNA regulatory networks in RMS. **Results**: We identified several lncRNAs with subtype-specific differential expression, including *HOTAIR* as a potential sponge for miR-206, *DSCR8* for miR-885-5p, and *PRKCQ-AS1* for miR-515-5p in eRMS. Database-supported interaction analyses identified putative regulatory relationships between these lncRNAs and cancer-related miRNAs and mRNAs. Validation using the St. Jude Cloud PeCan platform confirmed distinct lncRNA expression signatures across RMS subtypes and other pediatric solid tumors, supporting subtype-specific regulation. **Conclusions**: Our findings provide an updated characterization of the lncRNA landscape in RMS and identify candidate lncRNA–miRNA–mRNA regulatory networks that may contribute to disease biology. The proposed regulatory interactions are hypothesis-generating and require experimental validation. Overall, our findings provide a resource for future functional studies and support the investigation of lncRNAs as potential biomarkers and therapeutic targets in RMS.

## 1. Introduction

Rhabdomyosarcoma (RMS) is a malignant soft-tissue tumor arising from mesenchymal cells with skeletal muscle differentiation capability, RMS is characterized by its high aggressiveness, strong potential for local invasion and distant metastasis, and pronounced histological and molecular heterogeneity [1,2]. RMS typically originates in several tissues, such as the head and neck region, followed by the genitourinary tract, extremities, and chest [3]. RMS is the most common soft tissue sarcoma in childhood [4], accounting for 4 to 5% of all pediatric malignant tumors and representing the third most prevalent extracranial solid tumor in this age group in Brazil [5]. Its incidence is approximately 4.5 cases per million individuals under 20 years of age in the United States, and up to 4.9 cases per million in Sweden [6]. In some regions of Africa, the numbers are higher: in certain areas of Nigeria, the incidence is around 8 cases per million, reaching up to 16 cases per million in Malawi, in the eastern part of the continent [7]. Additionally, approximately 15–25% of cases are diagnosed already in a metastatic state, most commonly affecting the lungs, bone marrow, lymph nodes, and bones [5]. According to the World Health Organization (WHO), RMS is classified into four subtypes based on its clinicopathological and molecular genetic features: embryonal (eRMS), alveolar (aRMS), spindle cell/sclerosing (sRMS), and pleomorphic (pRMS), with the embryonal and alveolar forms being the most prevalent [8]. However, advances in molecular profiling have led to rapid refinements in RMS classification [9].

Beyond its clinical and histological heterogeneity, the molecular mechanisms underlying RMS remain poorly understood. In this context, noncoding RNAs have emerged as important regulators in cancer biology [10]. Among them, long noncoding RNAs (lncRNAs) have attracted increasing interest due to their roles in gene regulation and cellular differentiation. Although studies specifically addressing lncRNAs in RMS are still limited, numerous lncRNAs are known to regulate skeletal muscle differentiation, a process closely related to RMS pathogenesis. lncRNAs are transcripts longer than 200 nucleotides that do not encode proteins but may undergo splicing and polyadenylation, representing a substantial portion of the transcriptome [11,12]. Functionally, lncRNAs exhibit high cell type-specific expression and take part in diverse regulatory mechanisms, including chromatin remodeling, transcriptional control, and post-transcriptional regulation, such as acting as miRNA sponges [11,12]. MicroRNAs (miRNAs) are small noncoding RNAs that negatively regulate gene expression by promoting mRNA degradation or translational repression [13]. According to the competing endogenous RNA (ceRNA) hypothesis, lncRNAs can communicate with other RNA transcripts by competing for shared miRNAs through microRNA response elements (MREs), thereby modulating gene expression. Dysregulation of these ceRNA networks has been implicated in cancer development and progression, highlighting their potential relevance in RMS biology [13]. Despite their functional importance, the annotation and characterization of lncRNAs remain challenging due to their structural complexity and the substantial number of isoforms. Importantly, dysregulation of lncRNAs has been associated with a wide range of diseases, including cancer, reinforcing their potential relevance in RMS biology [11].

Despite the known characterization of lncRNAs in muscle differentiation and diseases such as cancer, their role in rhabdomyosarcoma remains poorly understood. Given the molecular heterogeneity and aggressive behavior of RMS, systematic investigation of lncRNA expression patterns and regulatory interactions is essential to elucidate mechanisms underlying tumor initiation and progression. Therefore, this study aims to perform a comprehensive characterization of lncRNA expression profiles across the two most prevalent RMS subtypes, embryonal and alveolar, and to delineate their associations with tumor subtype specification, disease progression, and putative molecular regulatory networks.

## 2. Methodology

### 2.1. Gene Expression Data

To better characterize the role of lncRNAs in RMS, we used the publicly available data from GSE28511, using platform GPL6947—Illumina Human HT-12 array (Li et al. [14], containing 23 samples, including 10 aRMS, 7 eRMS, and 6 non-tumor tissue controls (Supplementary Material S1).

We reannotated the GPL6947 probe annotations in R using Bioconductor resources. The original platform annotation table (GPL6947) was retrieved from the Gene Expression Omnibus (GEO) using the GEOquery package (v2.76.0). Updated gene annotations were obtained using the Bioconductor package illuminaHumanv4.db for probes with matching identifiers. Gene symbol, gene name, and Entrez Gene ID were extracted for each probe using the select() function and merged with the original platform annotation table based on probe identifiers. The resulting reannotated file was used for downstream analyses. A complete probe reannotation file, including the original GPL6947 annotations and the updated annotations for the lncRNAs identified in this study, is provided as Supplementary Material S2.

We processed the expression data in R (v4.5.1) using Biobase (v2.68.0), limma (v3.64.0), dplyr (v1.1.4), and the umap package (v0.2.10.0). Probes lacking valid gene annotation or mapping to multiple genes were removed. When multiple probes corresponded to the same gene, expression values were collapsed to gene level by retaining the probe with the highest variance across samples, resulting in a gene-level expression matrix.

We filtered the samples to exclude unclassified entries (“X”) and categorized into three groups: Control, eRMS, and aRMS. Expression values were log2-transformed when necessary to ensure appropriate normalization. The processed expression matrix, together with sample metadata and updated annotations, was organized into an ExpressionSet object.

We conducted differential expression analysis for both the original and reannotated datasets using linear models implemented in limma. A design matrix without an intercept was constructed, and contrasts were defined for eRMS vs. Control, aRMS vs. Control, and aRMS vs. eRMS. Linear model fitting was followed by empirical Bayes moderation.

Following statistical analysis, results were filtered to identify differentially expressed genes (DEGs) based on an absolute log2 fold change ≥ 1 and a false discovery rate (FDR) < 0.05. Genes with missing values were excluded during this step. Complete DEG tables and ranked results were exported for downstream analyses.

We performed quality assessment and visualization using boxplots, density plots, volcano plots, mean-difference (MD) plots, Venn diagrams, UMAP projections (n_neighbors = 10, random_state = 123), mean–variance trend plots, moderated t-statistic Q–Q plots, and adjusted *p*-value histograms, following a workflow analogous to GEO2R.

miRNA expression data were obtained from the Supplementary Data provided by Li, Sarver, Alamgir and Subramanian [14], generated using the Illumina Sentrix Array Matrix. Previously processed miRNA expression values from the “mean_all_miRNA_.txt” sheet of Supplementary Information S2 were used directly for the analysis. Because only processed expression values were publicly available, no additional background correction, normalization, or log2 transformation was performed. No samples or miRNAs were excluded prior to differential expression analysis. Differential expression analysis was performed using the limma package under the same statistical framework applied to lncRNAs and mRNAs; however, differentially expressed miRNAs were defined using an adjusted *p*-value < 0.05 and |log_2_FC| ≥ 2.

### 2.2. Gene Biotype Annotation

To characterize differentially expressed genes (DEGs) according to their biotype, we used R (v4.5.1) along with the biomaRt (v2.64.0) and dplyr (v1.1.4) packages. First, the DEG lists previously obtained for eRMS vs. Control and aRMS vs. Control were loaded.

Next, a connection to the Ensembl database was established using the biomaRt package with the *hsapiens_gene_ensembl* dataset. For each gene list, the gene symbol, Ensembl Gene ID, and corresponding biotype were retrieved using the getBM function, allowing classification of genes according to their functional characteristics. Based on the retrieved biotypes, categories of interest were defined: (1) lncRNAs, including the following Ensembl biotypes: lncRNA, lincRNA, antisense, antisense_RNA, bidirectional_promoter_lncRNA, macro_lncRNA, non_coding, processed_transcript, sense_intronic, sense_overlapping, 3prime_overlapping_ncRNA, TEC, retained_intron, and ambiguous_orf; and (2) mRNAs, including protein_coding, nonsense_mediated_decay, and non_stop_decay. DEGs were then filtered according to these categories, enabling separation between mRNAs and lncRNAs for each contrast (eRMS vs. Control and aRMS vs. Control). The results were exported as individual files, preserving the association of each gene with its biotype.

### 2.3. lncRNA Heatmap

We visualized differentially expressed lncRNAs in eRMS and aRMS in R using dplyr (v1.1.4), readr (v2.1.5), and pheatmap (v1.0.13). DEG files were read and filtered to retain *Gene.symbol* and *logFC*, with columns renamed to distinguish the subtypes. The tables were then merged by gene and converted into a matrix, with genes as rows and subtypes as columns.

### 2.4. lncRNA-miRNA-mRNA Interaction Networks

We constructed the lncRNA–miRNA–mRNA regulatory network based on the concept that lncRNAs can act as miRNA “sponges,” thereby indirectly modulating mRNA expression [15]. Networks were built in R using tidyverse (v2.0.0), igraph (v2.1.4), tidygraph (v1.3.1), ggraph (v2.2.2), and multiMiR (v1.30.0). Differentially expressed lncRNAs, miRNAs, and mRNAs were imported, standardized (gene symbols converted to uppercase), and filtered. To prioritize the most robust signals and reduce network complexity, mRNAs were further restricted to those with logFC threshold > 3. This threshold was applied solely for network construction and visualization, whereas differential expression analysis was performed using the predefined statistical criteria described above. Both validated and predicted lncRNA–miRNA and miRNA–mRNA interactions were retrieved using multiMiR. Edges were combined into directed graphs, and nodes were classified as lncRNA, miRNA, or mRNA. Networks were visualized using ggraph, and mRNAs included in each network were exported for downstream analyses, such as functional enrichment.

### 2.5. Enrichment Analysis

We performed KEGG enrichment analyses of the mRNAs interacting with miRNAs in the constructed lncRNA-miRNA-mRNA networks [15] using the ShinyGO 0.85 server [16] with default parameters.

### 2.6. Comparison of lncRNA Expression from Other Solid Tumors

To provide a complementary assessment of lncRNA differential expression between tumors, we used, via the PeCan platform, the St. Jude Cloud [17], an initiative of the *St. Jude Children’s Research Hospital*. This platform integrates genomic and expression data from approximately 9000 pediatric patients with hematologic tumors, central nervous system (CNS) tumors, and extracranial solid tumors, including both retrospective and prospective cohorts from reference institutions. Differentially expressed lncRNAs in RMS were queried using the platform’s expression tool, and their expression values, reported by the PeCan platform as log_10_(MoR + 1), were compared with those of other extracranial solid tumors. In this metric, MoR (Mean of Reads) represents the average normalized read count per gene, the addition of 1 avoids issues with zero values, and the log10 transformation reduces skewness and facilitates comparison across samples. This comparison provides contextual insight but should not be interpreted as a direct validation, since normal tissue data are not available in PeCan.

## 3. Results and Discussion

### 3.1. Re-Annotation

To better characterize the transcriptomic landscape of rhabdomyosarcoma (RMS), including the contribution of miRNAs and lncRNAs, we performed probe reannotation as described in Section 2. This approach enabled the identification of additional transcripts not captured in the original annotation, including 13 and 10 newly identified lncRNAs following probe reannotation in eRMS and aRMS, respectively.

### 3.2. Differential Expression Analysis of lncRNA, miRNA and mRNA

**Figure 1:**
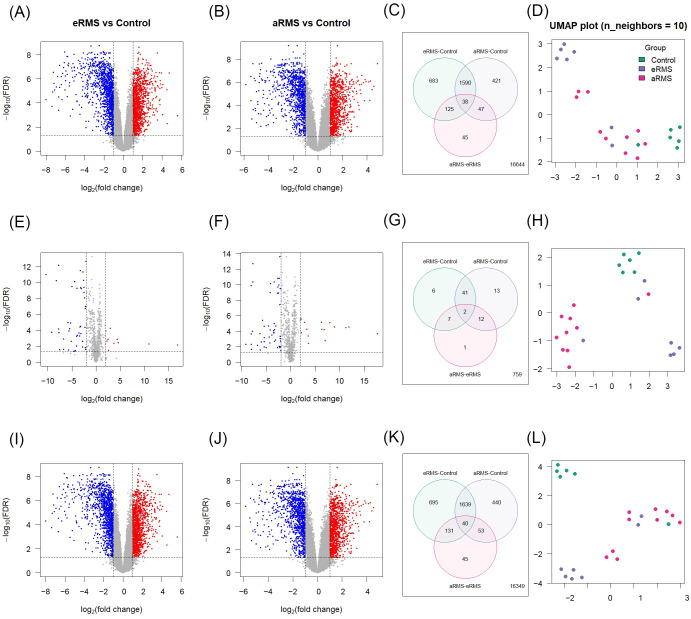
Volcano plots showing differentially expressed lncRNAs and mRNAs before probe reannotation (|log2 fold change| ≥ 1 and FDR < 0.05) in eRMS (**A**) and aRMS (**B**); differentially expressed miRNAs (|log2 fold change| ≥ 2 and FDR < 0.05) in eRMS (**E**) and aRMS (**F**); and differentially expressed lncRNAs and mRNAs after probe reannotation (|log2 fold change| ≥ 1 and FDR < 0.05) in eRMS (**I**) and aRMS (**J**). Upregulated DEGs are shown in red and downregulated in blue. Venn diagrams indicate the number of differentially expressed genes relative to control for lncRNAs and mRNAs before probe reannotation (**C**), for miRNAs (**G**), and for lncRNAs and mRNAs after probe reannotation (**K**). UMAP plots illustrate sample clustering based on lncRNA and mRNA expression before probe reannotation (**D**), miRNA expression (**H**), and lncRNA and mRNA expression after probe reannotation (**L**), highlighting the overall separation between eRMS (purple), aRMS (pink), and control samples (green).

To identify differentially expressed genes for eRMS and aRMS, we evaluated both the annotated vs. the re-annotated databases, finding that, when comparing eRMS versus control before probe reannotation, approximately 19,500 genes, including mRNAs and lncRNAs, were evaluated, of which ~2500 were identified as differentially expressed genes (DEGs) (|log2 fold change| ≥ 1 and FDR < 0.05) (Figure 1A). In the comparison of aRMS vs. Control, a similar number of genes (~19,500) were assessed, with ~2000 classified as DEGs (|log2 fold change| ≥ 1 and FDR < 0.05) (Figure 1B). The Venn diagram (Figure 1C) revealed that 16,644 genes were not differentially expressed between the groups. Among the DEGs, 1590 were shared between eRMS and aRMS, when comparing to the control group, suggesting potential common mechanisms across the subtypes. Additionally, 683 DEGs were unique to eRMS, 421 were unique to aRMS, and 45 were specific to the direct comparison between the subtypes, without differences relative to the control. The UMAP plot (Figure 1D) highlights the overall separation among eRMS, aRMS, and control samples.

In the comparison of eRMS vs. Control for miRNAs, we evaluated approximately 850 miRNAs, of which 57 were identified as DEG (|log2 fold change| ≥ 2 and FDR < 0.05) (Figure 1E). Similarly, in the comparison of aRMS vs. Control, ~850 miRNAs were assessed, with 69 classified as DEG (|log2 fold change| ≥ 2 and FDR < 0.05) (Figure 1F). The Venn diagram (Figure 1G) revealed that 759 miRNAs were not differentially expressed between the groups. Among the DEG miRNAs, 41 were shared between eRMS and aRMS relative to the control, suggesting potential common regulatory roles. Additionally, 6 DEG miRNAs were unique to eRMS, 13 were unique to aRMS, and 1 was specific to the direct comparison between the subtypes. The UMAP plot (Figure 1H) highlights the overall separation among eRMS, aRMS, and control samples based on miRNA expressions. Also, In the previous study, Li, Sarver, Alamgir and Subramanian [14] validated microarray findings using RT-qPCR and observed that miR-1 and miR-206 were significantly downregulated in RMS compared with controls, although these changes were not detected by microarray. Signal saturation in the microarray heatmaps likely masked the downregulation, which is why these miRNAs are also considered deregulated in the present study.

After probe reannotation, in the comparison of eRMS vs. Control, we evaluated approximately 19,300 genes, including mRNAs and lncRNAs, of which ~2500 were identified as differentially expressed genes (DEGs) (|log2 fold change| ≥ 1 and FDR < 0.05) (Figure 1I). In the comparison of aRMS vs. Control, a similar number of genes (~19,300) were assessed, with ~2100 classified as DEGs (|log2 fold change| ≥ 1 and FDR < 0.05) (Figure 1J). The Venn diagram (Figure 1K) revealed that 16,349 genes were not differentially expressed between the groups. Among the DEGs, 1639 were shared between eRMS and aRMS relative to the control, suggesting potential common mechanisms across the subtypes. Additionally, 695 DEGs were unique to eRMS, 440 were unique to aRMS, and 45 were specific to the direct comparison between the subtypes. The UMAP plot (Figure 1L) highlights the overall separation among eRMS, aRMS, and control samples after probe reannotation.

We performed additional quality assessments, including the MD plot, boxplots, expression density plots, adjusted *p*-value histogram, moderated t-statistic Q-Q plot, and mean-variance trend. These analyses are provided in Supplementary Material S3.

Our study revealed that the eRMS subtype shows a significantly higher number of differentially expressed genes compared to alveolar aRMS. This finding can be attributed to the distinct molecular mechanisms that characterize each subtype, reflecting their cellular origins and tumor evolution pathways. eRMS is typically “fusion-negative,” lacking oncogenic fusions such as PAX3-FOXO1 [18], which may account for its more heterogeneous gene expression landscape and the greater variability observed in genes regulating muscle differentiation, proliferation, and response to external stimuli.

### 3.3. Comparison of lncRNA Differential Expression in eRMS and aRMS Before and After Probe Reannotation, lncRNA-miRNA-mRNA Network and Enrichment Analyses

**Figure 2:**
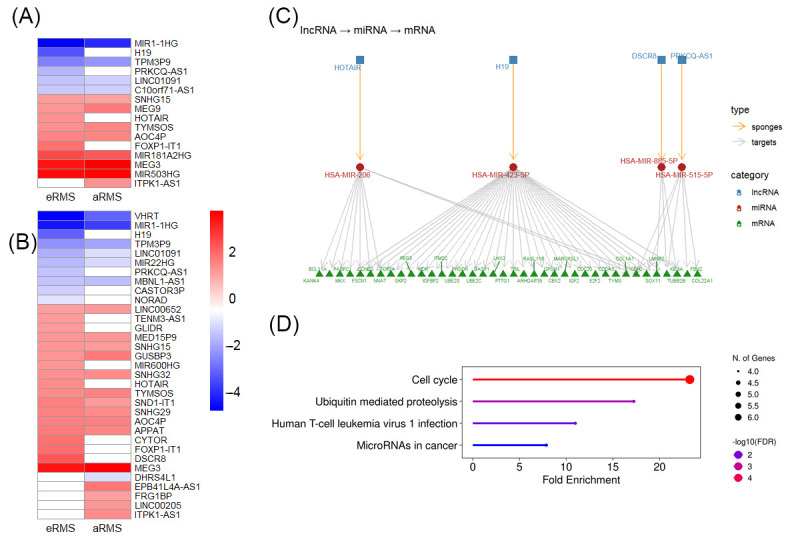
Comparison of lncRNA differential expression in eRMS and aRMS before probe reannotation (**A**) and after reannotation (**B**) demonstrates an increase in the number of detected differentially expressed genes (DEGs) following reannotation. The heatmap scale represents log_2_ fold change (logFC) of the DEGs. The lncRNA-miRNA-mRNA interaction network analysis for eRMS is shown in (**C**), based on the DEG lists. No interaction networks could be constructed for aRMS. Enrichment analyses of mRNAs associated with miRNAs in the eRMS networks after reannotation (**D**).

Before probe reannotation, we identified 15 of the ~2500 DEGs in eRMS as lncRNAs, while 12 of the ~2000 DEGs in aRMS were classified as lncRNAs (Figure 2A). After probe reannotation, the number of lncRNAs increased to 28 of the ~2500 DEGs in eRMS and 22 of the ~2100 DEGs in aRMS (Figure 2B).

No network could be constructed for aRMS. For eRMS, the lncRNA-miRNA-mRNA network, constructed based on the concept that lncRNAs can indirectly interact with mRNAs by acting as miRNA “sponges” and thereby regulating mRNA activity [15], identified *HOTAIR* as a potential sponge for miR-206, H19 for miR-423-5p, *DSCR8* for miR-885-5p, and *PRKCQ-AS1* for miR-515-5p in eRMS (Figure 2C).

KEGG enrichment analysis of highly expressed mRNAs (logFC > 3) interacting with miRNAs in the lncRNA-miRNA-mRNA network revealed that, in eRMS, the top pathways included cell cycle regulation, ubiquitin-mediated proteolysis, viral infections, microRNAs in cancer (Figure 2D). These findings suggest that the mRNAs interacting with miRNAs may participate in critical processes related to RMS development and progression.

Lu et al. [19] identified 498 up- and 480 down-regulated DEGs, with top KEGG pathways including cell cycle, cellular senescence, p53, FoxO signaling, and ubiquitin-mediated proteolysis. These results support the relevance of cell cycle and ubiquitin-mediated proteolysis pathways, which were also enriched in our analysis of mRNAs interacting with the miRNAs from the network analysis, suggesting their potential role in RMS development.

Building on these insights into RMS-related pathways, we next focused on evaluating lncRNAs associated with the disease, using the results obtained following probe re-annotation to guide our analysis. Performing an accurate, comprehensive, and up-to-date re-annotation of microarray probes substantially improves the reliability of analyses using Illumina platforms [20]. As previously discussed, this approach not only enhances the precision of new experiments but also enables the re-evaluation of previously published datasets, thereby expanding both the scope and depth of biological insights that can be obtained from BeadArray experiments [20].

To facilitate the interpretation of the main findings, Table 1 summarizes the principal lncRNAs identified in this study, their differential expression patterns in RMS, putative biological relevance, and the current evidence available in RMS and other cancer types. This overview provides a framework for the subsequent discussion while emphasizing that the proposed regulatory roles are based on the available literature and bioinformatic analyses and therefore require experimental validation.

### 3.4. Putative lncRNA–miRNA Sponge Interactions Potentially Involved in RMS Regulation

**Figure 3:**
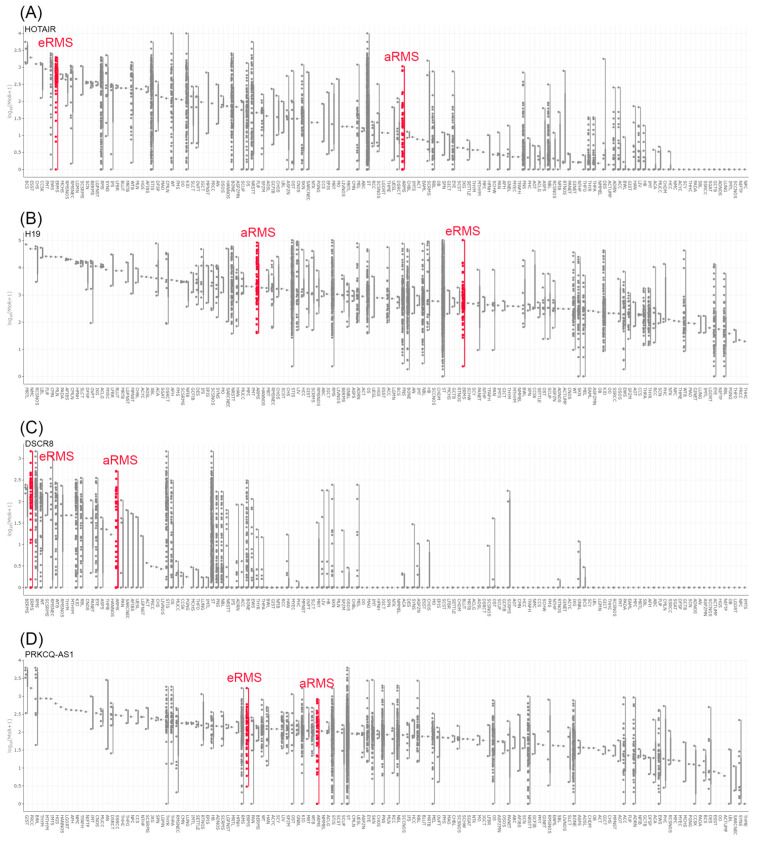
Comparison of the expression of the lncRNAs observed regulated in this study with their expression profiles in other solid tumors from PeCan platform. (**A**) Results for *HOTAIR*. (**B**) Results for *H19*. (**C**) Results for *DSCR8*. (**D**) Results for *PRKCQ-AS1*. Gene expression represented by log_10_(MoR + 1) values. MoR = median of ratios.

Considering the lncRNA–miRNA–mRNA network analysis, we identified *HOTAIR*, *H19*, *DSCR8* and *PRKCQ-AS1* as possible lncRNAs that may act as miRNA sponges in eRMS. These database-supported associations have not been experimentally validated in rhabdomyosarcoma and should therefore be considered hypothesis-generating. Comparing the expression of these lncRNAs observed regulated in this study: *HOTAIR* (logFC = +1.36), *H19* (logFC = −3.17), *DSCR8* (logFC = +2.20) e *PRKCQ-AS1* (logFC = −1.57), with their expression profiles in other solid tumors from PeCan platform (Figure 3A–D), it becomes evident that *HOTAIR* is more highly expressed in eRMS compared with aRMS and other cancer types (Figure 3A).

*H19* expression was significantly reduced in RMS tissues, particularly in eRMS, compared with normal muscle [21], as we also observed in our study (eRMS: logFC = −3.17; no significant change in aRMS). This is further supported by the comparison of expression profiles across other solid tumors on the PeCan platform (Figure 3B), which shows markedly lower *H19* levels in eRMS compared with aRMS and other solid tumors.

*DSCR8* is upregulated in other cancers, such as hepatocellular carcinoma [22] and ovarian cancer [23], although no studies have reported its role in RMS. Comparison of expression profiles across other solid tumors using the PeCan platform (Figure 3C) revealed high *DSCR8* expression in RMS relative to other tumors, with eRMS showing higher expression than aRMS, corroborating the findings of this study.

Comparison of expression profiles across other solid tumors using the PeCan platform (Figure 3D) revealed that *PRKCQ-AS1* displays moderately high expression compared to other tumors and higher levels in eRMS than in aRMS, which contrasts with the results obtained in the present study. Furthermore, several studies describe *PRKCQ-AS1* as having an oncogenic function, as reported in colorectal cancer [24] and neuroblastoma [25]. However, others describe as a suppressor, as reported for lung adenocarcinoma [26]. This discrepancy may be related to the potential dual roles of this lncRNA.

*HOTAIR* has been described as an endogenous “sponge,” reducing some miR expression by directly binding to it [27]. Furthermore, *HOTAIR* was shown to reverse the effects of miR-1 on proliferation and cell cycle progression in esophageal squamous cell carcinoma (ESCC) cells by derepressing CCND1, a direct target of miR-1 [28]. Since miR-1 is also downregulated in RMS, contributing to tumor progression, understanding the mechanisms underlying this reduction is crucial. One possible contributor is the lncRNA *HOTAIR*, which may play a key role in regulating miR-1 expression and thereby promoting oncogenic pathways in eRMS. Furthermore, based on the database-supported lncRNA–miRNA–mRNA network analysis, the upregulated lncRNA *HOTAIR* is predicted to interact with miR-206, which is downregulated in RMS, suggesting a potential sponge-mediated regulatory mechanism. Similar regulatory mechanisms have been reported in other cancer types, including colorectal cancer 26, head and neck squamous cell carcinoma 27, medulloblastoma 28, and breast cancer 29. Similar regulatory mechanisms have been reported in other cancer types, including colorectal cancer [29], head and neck squamous cell carcinoma [30], medulloblastoma [31], and breast cancer [32].

The lncRNA *H19* was one of the first lncRNAs to have its function described in different biological contexts [10]. miR-423-5p (eRMS: logFC = −2.78) has been reported to be downregulated in several types of cancer, as ovarian cancer [33], esophageal squamous cell [34], and gastric cancer [35]. Therefore, *H19* might contribute to RMS progression by blocking the tumor-suppressive effects of miR-423-5p. Instead of implying a classical sponge mechanism, we can highlight that although *H19* was a documented sponge for miR-423-5p [36], its downregulation in eRMS suggests that alternative regulatory mechanisms may be at play in this context. *H19* exhibits dual functions in cancer, acting both as an oncogene and as a tumor suppressor through distinct mechanisms [10]. Additionally, it is also described as a precursor for miR-675, located in its first exon; reactivation of *H19* in RMS cells was shown to inhibit tumor growth by increasing miR-675 expression and promoting muscle differentiation [10].

*DSCR8* may act as an oncogenic lncRNA by sponging miR-885-5p (eRMS: logFC = −10.38), which was identified as a potential interacting miRNA in the network analysis, thereby preventing its tumor-suppressive effects. Indeed, miR-885-5p has been described as a tumor suppressor that promotes the accumulation of p53 protein and activates the p53 pathway, leading to the upregulation of p53 target genes in neuroblastoma cell lines [37]. *DSCR8* has also been reported to promote liver cell proliferation and inhibit apoptosis by regulating miR-22-3p [38]. A similar mechanism may occur in RMS, as we observed upregulation of *DSCR8* in eRMS (logFC = +2.20; no significant change in aRMS) accompanied by downregulation of miR-22 (eRMS: logFC = −2.70; aRMS: logFC = −2.34).

Given that miR-515-5p shows comparable downregulation in both RMS subtypes (eRMS: logFC = −5.82; aRMS: logFC = −5.75), it is unlikely that *PRKCQ-AS1* plays a major role in its regulation, suggesting that other mechanisms may be involved. The function of this lncRNA in RMS remains to be elucidated, and it may participate in alternative regulatory pathways unrelated to miR-515-5p.

Considering the literature, several lncRNAs identified as differentially regulated in the present study have previously been reported to function as competing endogenous RNAs (ceRNAs) through miRNA sponging in other cancer types, including *GLIDR*, *LINC00205*, *MBNL1-AS1*, *MIR600HG*, *NORAD*, *SND1-IT1*, *TYMSOS,* members of the *SNHG* family, *APPAT*, *CYTOR*, and *EPB41L4A-AS1*.

*GLIDR*, which was upregulated in eRMS (logFC = +1.11) but not significantly altered in aRMS, has been shown to promote invasion, epithelial–mesenchymal transition (EMT), and proliferation in prostate cancer cells by sponging miR-128-3p. This interaction reverses miR-128-3p–mediated repression of EMT markers, whereas *GLIDR* knockdown suppresses these oncogenic processes [39]. In addition, *GLIDR* is upregulated in lung adenocarcinoma, where it enhances tumor cell survival by sponging miR-1270, thereby derepressing TCF12 and promoting proliferation while inhibiting apoptosis [40].

*LINC00205*, which was upregulated in aRMS (logFC = +1.18) but unchanged in eRMS, is overexpressed in gastric cancer and associated with poor prognosis. Functionally, *LINC00205* acts as a ceRNA for miR-26a, leading to derepression of oncogenic targets such as HMGA2, EZH2, and USP15, thereby facilitating EMT and tumor progression [41].

*MBNL1-AS1* was downregulated in both eRMS (logFC = −1.55) and aRMS (logFC = −1.62). Consistently, *MBNL1-AS1* has been described as a tumor suppressor in breast cancer, where it inhibits proliferation, migration, invasion, and glycolysis by sponging miR-889-3p and restoring expression of the tumor suppressor KLF9 [42]. Similarly, in non-small cell lung cancer, *MBNL1-AS1* suppresses tumor progression by binding miR-135a-5p, with in vivo studies confirming its inhibitory effects on tumor growth [43].

*MIR600HG*, which was upregulated in eRMS (logFC = +1.22), has been reported to promote colorectal cancer progression, with high expression correlating with increased tumor size and advanced TNM stage. Mechanistically, *MIR600HG* regulates downstream targets involving the miR-144-3p/KIF3A axis [44].

*NORAD*, downregulated in eRMS (logFC = −1.07), functions as a tumor suppressor in hepatocellular carcinoma by sponging miR-106a-5p and preserving PTEN expression [45]. A similar tumor-suppressive role has been described in breast cancer, where *NORAD* inhibits proliferation and invasion through the miR-155-5p/SOCS1 axis [46].

*SND1-IT1* was upregulated in both eRMS (logFC = +1.49) and aRMS (logFC = +1.11). In gastric cancer, *SND1-IT1* promotes TGF-β1–induced EMT by sponging miR-124 and upregulating COL4A1 [47]. In retinoblastoma, *SND1-IT1* enhances tumor growth and invasiveness through the miR-132-3p/SMAD2 axis, correlating with poor patient prognosis [48].

*TYMSOS*, upregulated in both eRMS (logFC = +1.39) and aRMS (logFC = +1.54), has been implicated in cervical squamous cell carcinoma, where it promotes proliferation, invasion, and immune escape by sponging miR-134-5p and upregulating KRAS, thereby impairing NK cell–mediated cytotoxicity [49].

Members of the *SNHG* family, including *SNHG15*, *SNHG29*, and *SNHG32*, were consistently upregulated in both RMS subtypes. High *SNHG* expression has been associated with poor survival in cervical cancer [50], and several studies suggest that *SNHG*-mediated oncogenic effects may involve miRNA sponging mechanisms [51,52,53,54]. However, no overlap was observed between previously reported SNHG target genes and those differentially expressed in the present RMS dataset.

*APPAT* was upregulated in both eRMS (logFC = +1.61) and aRMS (logFC = +1.46). Although *APPAT* has been shown to regulate breast cancer progression via the miR-328a/Pkp1 pathway [55], neither miR-328a nor Pkp1 were differentially expressed in RMS, suggesting that *APPAT* may act through alternative mechanisms in this context.

*CYTOR* was upregulated in eRMS (logFC = +1.75) and has been associated with advanced tumor stages across multiple cancer types [56]. *CYTOR* has been reported to sponge miR-206 in pituitary tumor cells [57]. Although this interaction was not detected in our network analysis, CYTOR may exert similar regulatory effects in RMS.

Finally, *EPB41L4A-AS1*, which was upregulated in aRMS (logFC = +1.67), has been implicated in gastric cancer and non-small cell lung cancer through miRNA-mediated mechanisms [58,59]. However, neither the reported miRNA nor mRNA targets were differentially expressed in RMS, indicating that *EPB41L4A-AS1* may operate via distinct regulatory pathways in this tumor context.

### 3.5. lncRNAs with miRNA Host Function Involved in RMS Regulation

Although most miRNAs are intronic to protein-coding genes, many originate from lncRNAs whose processing differs from canonical mRNA maturation [60]. MIR1-1HG, and *MIR22-HG* are examples of host genes that were observed as regulated in RMS in this study.

*MIR1-1HG* was differentially expressed in both eRMS (logFC = −4.48) and aRMS (logFC = −3.80). Li, Sarver, Alamgir and Subramanian [14] observed by RT-qPCR that, compared with the control group, miR-1 expression levels were decreased. The authors reported that “signal intensity analyses for the microarray heatmap revealed that signals from miR-1 were highly saturated in both control and RMS tissue samples”, which interfered with the expression analysis.

**Figure 4:**
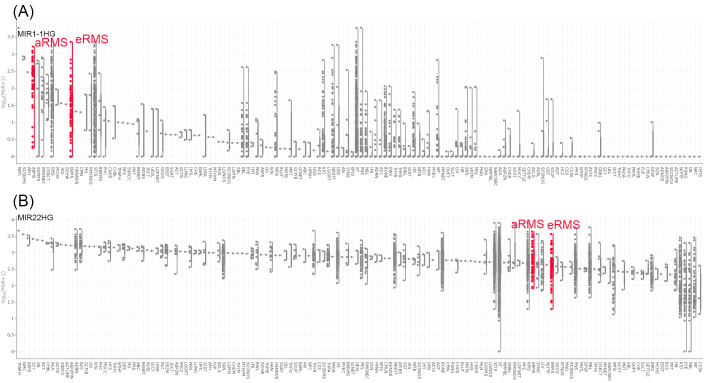
Comparison of the expression of the lncRNAs observed regulated in this study with their expression profiles in other solid tumors from PeCan platform. (**A**) Results for *MIR1-1HG*. (**B**) Results for *MIR22HG*. Gene expression represented by log_10_(MoR + 1) values. MoR = median of ratios.

Indeed, when we compared the expression of the precursor lncRNA *MIR1-1HG* (also reported as the miR-133a-2 host gene in some references [61,62]), identified as regulated in our study, with their expression profiles across other solid tumors in the PeCan platform (Figure 4A), we observed that its levels were markedly higher compared with other solid tumors, further supporting the observations reported by Li, Sarver, Alamgir and Subramanian [14]. Previous studies have shown that miR-1 expression is induced during human muscle cell differentiation and progressively increases with advancing stages of fetal muscle development [63].

*MIR22-HG* was differentially expressed in both eRMS (logFC = −1.73) and aRMS (logFC = −1.21). When compared with expression profiles across other solid tumors on the PeCan platform (Figure 4B), we also observed a substantial reduction in this lncRNA relative to other solid tumors.

The lncRNA *MIR1-1HG* serves as the host gene for miR-1 [61,62]. Both miR-1 and miR-206, which differ by only three nucleotides, have binding sites within the 3′UTR region of *CCND2*, and ectopic expression of either miRNA was shown to downregulate *CCND2* levels in RMS cells, as observed in the study that generated the dataset used here [14]. CCND2 has been identified as a key regulator promoting myogenic differentiation of muscle progenitor cells in dystrophin-deficient mice [64]. Moreover, RMS cells display elevated expression of cell cycle regulatory genes, including CCND2, compared with normal skeletal myocytes [65].

The lncRNA *MIR22-HG*, which serves as the host gene for miR-22 [66], was also found to be downregulated in the present study. miR-22, normally induced during muscle differentiation, is reduced in RMS, and its restoration has been shown to block tumor growth and dissemination in in vivo models [67]. Moreover, miR-22 is downregulated in multiple cancer types, including hepatocellular carcinoma, esophageal squamous cell carcinoma, colorectal cancer, and gastric cancer [68,69,70,71]. Qiao et al. [72] identified a novel miR-22-3p/ENO1 regulatory axis involved in gastric cancer development, and miR-22-3p has also been reported to promote gastric cancer progression via the BCL9–Wnt/β-catenin signaling pathway [73]. Since both *ENO1* (eRMS: logFC = +1.97; aRMS logFC = +1.66) and *BCL9* (eRMS: logFC = +1.07; no significant change in aRMS) were found to be upregulated in the present study, these pathways may likewise be affected by miR-22 in RMS.

### 3.6. lncRNAs with Differential Mechanisms in RMS Compared to Other Cancers

**Figure 5:**
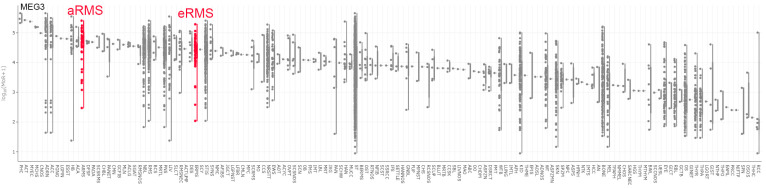
Comparison of the expression of the lncRNAs *MEG3*, observed regulated in this study with their expression profiles in other solid tumors from PeCan platform. Gene expression represented by log_10_(MoR + 1) values. MoR = median of ratios.

*MEG3* has been found to be downregulated in a wide range of neoplasms, including cancers of the digestive system (esophageal squamous cell carcinoma, gastric cancer, hepatocellular carcinoma, colorectal cancer, pancreatic cancer, cholangiocarcinoma, gallbladder cancer), urogenital cancers (renal cell cancer, prostate cancer, testicular germ cell tumor, bladder cancer, ovarian cancer, endometrial cancer), nervous system tumors (neuroblastoma, glioma, meningioma, retinoblastoma), bone and soft tissue tumors (osteosarcoma, chordoma), and other malignancies such as squamous cell carcinoma of the head and neck, breast cancer, cervical cancer, choriocarcinoma, leukemia, multiple myeloma, T-cell lymphoblastic lymphoma, thyroid cancer, and melanoma [74]. However, in our study, we observed a significant upregulation of *MEG3* in both eRMS (logFC = +3.32) and aRMS (logFC = +3.70). When compared with expression profiles across other solid tumors on the PeCan platform, *MEG3* is also upregulated relative to these tumors (Figure 5).

A decrease in *MEG3* expression with age was observed in quadriceps muscle of older male mice (28 months vs. 12 weeks) [75]. Prolonged suppression of *MEG3* in C2C12 myoblasts promoted epithelial-to-mesenchymal transition (EMT) and impaired essential cellular transitions required for differentiation [76]. *MEG3* knockdown compromised muscle regeneration, resulting in abnormal mesenchymal gene expression and increased interstitial proliferation. Transcriptomic analysis revealed dysregulation of EMT-related genes, with *TGF-β* identified as a key regulator. Inhibition of TGF-βR1, its downstream effectors, and the transcription factor SNAI2 was able to restore myogenic differentiation. Moreover, decreased Ezh2 activity dependent on *MEG3* caused epigenetic changes linked to TGF-β activation. Thus, Dill, Carroll, Pinheiro, Gao and Naya [76] concluded that *MEG3* is essential for maintaining myoblast identity and promoting progression of muscle differentiation.

Considering that RMS originates from muscle progenitor or intermediate cells, high *MEG3* expression may: (1) reflect its physiological function in muscle progenitors or mesenchymal stem cells, maintaining cellular identity and plasticity; (2) contribute to blocking terminal differentiation, keeping cells in an immature state adaptive to the tumor environment; and (3) diverge from its classical tumor suppressor role in other cancers, reinforcing the concept of context- and cell type-dependent functions.

The *CASTOR3P* (*GATS*) gene has been reported to suppress breast cancer cell proliferation by inducing autophagy through inhibition of the PI3K/Akt signaling pathway [77]. This tumor-suppressive role contrasts with the findings of the present study, in which *CASTOR3P* was downregulated in eRMS (logFC = −1.41) and not significantly altered in aRMS. To date, there is no evidence supporting an oncogenic role for *CASTOR3P* in other cancer types, suggesting that its functional relevance in RMS remains unclear.

Tumor-derived exosomal *LINC01091*, which was downregulated in both eRMS (logFC = −1.90) and aRMS (logFC = −1.17), has been shown to promote gastric cancer progression by acting as a sponge for miR-128-3p, thereby derepressing ELF4 and transcriptionally activating CDX2. Silencing exosomal *LINC01091* suppresses gastric cancer cell proliferation, migration, invasion, and tumor growth by disrupting the miR-128-3p/ELF4/CDX2 regulatory axis [78].

Finally, the lncRNA-derived microprotein *TPM3P9*, markedly upregulated in eRMS (logFC = +2.42) and aRMS (logFC = +2.04), has been implicated in clear cell renal cell carcinoma progression through modulation of oncogenic RNA splicing. *TPM3P9* binds RBM4, inhibits RBM4-mediated exon skipping of TCF7L2, and promotes expression of the oncogenic splice variant TCF7L2-L, which activates NF-κB signaling via RELB induction. Clinically, high *TPM3P9* expression or low RBM4 levels are associated with poor patient survival, highlighting *TPM3P9* as a potential prognostic marker and therapeutic target in ccRCC [79].

### 3.7. lncRNAs Associated with Cancer Prognosis and Putative Biological Functions

*VHRT* (*LINC01405*) was markedly downregulated in both eRMS (logFC = −4.85) and aRMS (logFC = −3.21). Consistently, *VHRT* has also been reported as significantly downregulated in esophageal squamous cell carcinoma compared with adjacent non-tumor tissues, suggesting a conserved potential tumor-suppressive role across distinct cancer types [80].

*AOC4P*, upregulated in both eRMS and aRMS (logFC = +1.56), has been shown to be overexpressed in gastric cancer, where high expression correlates with poor overall survival and lymphovascular invasion [81]. Functional studies demonstrated that *AOC4P* knockdown suppresses cancer cell proliferation, migration, and invasion, induces apoptosis, and reduces tumor growth while modulating epithelial–mesenchymal transition (EMT) markers, underscoring its oncogenic role in gastric cancer progression [81].

Reduced expression of *FOXP1-IT1* has been reported in gastric cancer as well as in ovarian cancer stroma and epithelial tissues, although the underlying mechanisms remain unclear [82,83]. In contrast, *FOXP1-IT1* was upregulated in eRMS samples (logFC = +1.87), suggesting a potential context-dependent or lineage-specific oncogenic role in RMS.

Alterations in *FRG1BP*, which showed no significant change in eRMS but was downregulated in aRMS (logFC = −1.03), have been associated with prostate adenocarcinoma [84] and with colorectal cancer within one of the seven molecular subtypes described by Dashti et al. [85]. However, its functional contribution to RMS biology remains largely unexplored.

*ITPK1-AS1*, upregulated in aRMS (logFC = +1.39), has been identified as a prognosis-associated lncRNA in gastric cancer and proposed as a potential biomarker linked to disease pathogenesis and patient outcome [86].

*LINC00652* was modestly upregulated in both eRMS (logFC = +1.03) and aRMS (logFC = +1.07). Consistently, this lncRNA has been reported as significantly upregulated in breast cancer samples from Isfahan patients, where its expression correlated with clinicopathological features, and ROC analysis supported its potential diagnostic and prognostic value (preprint) [87].

Finally, *TENM3-AS1*, upregulated in eRMS (logFC = +1.11), has been implicated in gastric cancer metastasis and advanced disease stages, where high expression is associated with poor survival [88]. Functionally, *TENM3-AS1* promotes cancer cell migration, invasion, tumor growth, and liver metastasis by enhancing fatty acid biosynthesis. Mechanistically, it is transcriptionally activated by EGR1 and interacts with hnRNPK, stabilizing *FASN* mRNA and driving metabolic reprogramming through the EGR1/TENM3-AS1/hnRNPK/FASN axis, highlighting *TENM3-AS1* as a potential therapeutic target [88].

### 3.8. lncRNAs with Limited Functional Characterization in Cancer

*DHRS4L1* showed no significant change in eRMS and was downregulated in aRMS (logFC = −1.09). To date, no direct association between *DHRS4L1* and cancer has been reported. The human *DHRS4* gene cluster comprises *DHRS4*, *DHRS4L2*, and the recently annotated *DHRS4L1*. However, limited information is available regarding the structure, regulation, or biological function of *DHRS4L1*, and its role in cancer biology remains largely unexplored [89].

Similarly, no association with cancer has been described for *MED15P9*, despite its upregulation in both eRMS (logFC = +1.17) and aRMS (logFC = +1.15). The absence of functional or mechanistic studies suggests that the potential contribution of *MED15P9* to tumorigenesis remains unknown.

## 4. Final Comments

In summary, our study provides an updated characterization of the lncRNA landscape in rhabdomyosarcoma through the reannotation of publicly available transcriptomic data. We identified differentially expressed lncRNAs, including *HOTAIR*, *H19*, *MIR1-1HG*, *MEG3*, *DSCR8*, and *PRKCQ-AS1*, and explored their putative regulatory relationships with miRNAs and mRNAs using database-supported interaction networks. These findings identify candidate lncRNA–miRNA–mRNA regulatory networks that may contribute to RMS biology through competing endogenous RNA (ceRNA)-mediated mechanisms. Depending on their molecular targets and cellular context, lncRNAs may function as oncogenes or tumor suppressors by modulating the availability of miRNAs and, consequently, the expression of their target genes. However, the proposed regulatory interactions were inferred from bioinformatic analyses and literature-supported databases, and their functional relevance in rhabdomyosarcoma remains to be experimentally validated. Overall, our findings provide a resource for future mechanistic studies and support the investigation of lncRNAs as potential biomarkers and therapeutic targets in RMS.

## 5. Study Limitations and Future Perspectives

The present study has some limitations. The differential expression analyses were based on a single publicly available transcriptomic dataset with a limited sample size. To provide complementary support for the main findings, the expression patterns of the candidate lncRNAs were compared using the independent pediatric cancer resource available through the PeCan platform. However, this comparison should not be considered a direct validation because normal tissue data are not available in PeCan. In addition, although GSE28511 includes basic clinicopathological information, it does not provide overall survival, therapeutic response, recurrence, or follow-up data, precluding analyses of the prognostic and clinical significance of the identified lncRNAs. Furthermore, the proposed lncRNA–miRNA–mRNA interactions are based on database-supported predictions and should be considered hypothesis-generating until experimentally validated in rhabdomyosarcoma. Future studies should validate these candidate lncRNAs and their predicted regulatory interactions in independent, clinically annotated cohorts and through functional experiments to determine their biological roles and potential clinical utility in rhabdomyosarcoma.

## Figures and Tables

**Table 1:** Summary of the lncRNAs identified in rhabdomyosarcoma and their putative biological relevance.

lncRNA	Expression in RMS	Category	Main Finding in This Study	Evidence in RMS	Evidence in Other Cancers
*HOTAIR*	↑ eRMS	Putative ceRNA	Predicted interaction with miR-206	No	Yes
*H19*	↓ eRMS	Putative ceRNA	Predicted interaction with miR-423-5p; possible context-dependent mechanism	Yes	Yes
*DSCR8*	↑ eRMS	Putative ceRNA	Predicted interaction with miR-885-5p	No	Yes
*PRKCQ-AS1*	↓ eRMS	Putative ceRNA	Predicted interaction with miR-515-5p; limited support in RMS	No	Yes
*GLIDR*	↑ eRMS	Putative ceRNA	Previously reported ceRNA in other cancers	No	Yes
*LINC00205*	↑ aRMS	Putative ceRNA	Previously reported ceRNA in gastric cancer	No	Yes
*MBNL1-AS1*	↓ eRMS, ↓ aRMS	Putative ceRNA	Reported tumor suppressor in several cancers	No	Yes
*MIR600HG*	↑ eRMS	Putative ceRNA	Reported regulator of the miR-144-3p/KIF3A axis	No	Yes
*NORAD*	↓ eRMS	Putative ceRNA	Reported tumor suppressor in liver and breast cancer	No	Yes
*SND1-IT1*	↑ eRMS, ↑ aRMS	Putative ceRNA	Associated with EMT and tumor progression	No	Yes
*TYMSOS*	↑ eRMS, ↑ aRMS	Putative ceRNA	Reported oncogenic ceRNA	No	Yes
*SNHG15*	↑ eRMS, ↑ aRMS	Putative ceRNA	Member of oncogenic *SNHG* family	No	Yes
*SNHG29*	↑ eRMS, ↑ aRMS	Putative ceRNA	Member of oncogenic *SNHG* family	No	Yes
*SNHG32*	↑ eRMS, ↑ aRMS	Putative ceRNA	Member of oncogenic *SNHG* family	No	Yes
*APPAT*	↑ eRMS, ↑ aRMS	Putative ceRNA	No overlap with reported downstream targets in RMS	No	Yes
*CYTOR*	↑ eRMS	Putative ceRNA	Previously reported miR-206 sponge	No	Yes
*EPB41L4A-AS1*	↑ aRMS	Putative ceRNA	Previously associated with miRNA-mediated regulation	No	Yes
*MIR1-1HG*	↓ eRMS, ↓ aRMS	miRNA host gene	Host gene of miR-1; associated with myogenic differentiation	Yes	Yes
*MIR22HG*	↓ eRMS, ↓ aRMS	miRNA host gene	Host gene of miR-22	No	Yes
*MEG3*	↑ eRMS, ↑ aRMS	Context-dependent	Opposite expression pattern compared with most cancers	No	Yes
*CASTOR3P*	↓ eRMS	Context-dependent	Putative lineage-specific role in RMS	No	Limited
*LINC01091*	↓ eRMS, ↓ aRMS	Context-dependent	Opposite behavior compared with gastric cancer	No	Yes
*TPM3P9*	↑ eRMS, ↑ aRMS	Context-dependent	Encodes a microprotein implicated in oncogenic splicing	No	Yes
*VHRT (LINC01405)*	↓ eRMS, ↓ aRMS	Prognostic candidate	Potential tumor suppressor	No	Yes
*AOC4P*	↑ eRMS, ↑ aRMS	Prognostic candidate	Associated with poor prognosis in gastric cancer	No	Yes
*FOXP1-IT1*	↑ eRMS	Prognostic candidate	Possible context-dependent oncogenic role	No	Yes
*FRG1BP*	↓ aRMS	Prognostic candidate	Function in RMS remains unknown	No	Limited
*ITPK1-AS1*	↑ aRMS	Prognostic candidate	Prognostic biomarker in gastric cancer	No	Yes
*LINC00652*	↑ eRMS, ↑ aRMS	Prognostic candidate	Potential diagnostic/prognostic biomarker	No	Yes
*TENM3-AS1*	↑ eRMS	Prognostic candidate	Associated with metastasis and poor prognosis	No	Yes
*DHRS4L1*	↓ aRMS	Poorly characterized	No functional evidence in cancer	No	No
*MED15P9*	↑ eRMS, ↑ aRMS	Poorly characterized	Biological role remains unknown	No	No

↑ indicates upregulation; ↓ indicates downregulation.

## Data Availability

The original contributions presented in this study are included in the article/Supplementary Material. Further inquiries can be directed to the corresponding authors.

## Supplementary Materials

The following supporting information can be downloaded at: https://www.mdpi.com/article/10.3390/biomedicines14071648/s1, Supplementary Material S1: Information on the samples used in the study; Supplementary Material S2: Original GPL6947 probe annotations and updated probe annotations generated using the illuminaHumanv4.db Bioconductor package for the lncRNAs identified; Supplementary Material S3: Figure S1. Differential expression analysis and quality assessment.
