# Supplementary material for: Reannotation of Public Transcriptomic Data Identifies Candidate lncRNAs and Putative Regulatory Networks in Rhabdomyosarcoma
Source: Biomedicines. 2026 Jul 22;14(7):1648. doi: 10.3390/biomedicines14071648 (PMC13406521; doi:10.3390/biomedicines14071648)
Supplement: Supplementary file 1 [file biomedicines-14-01648-s001.zip › Supplementary Material S1.pdf]

Supplementary Material S1. Information on the samples used in the study. The original study analyzed a total of 48 tumor and normal tissue samples; however, not all samples are available in the GEO database. To better characterize the role of lncRNAs in rhabdomyosarcoma (RMS), publicly available data from GSE28511 (platform GPL6947 – Illumina Human HT-12 array) were used, comprising 23 samples: 10 alveolar RMS (aRMS), 7 embryonal RMS (eRMS), and 6 non-tumor tissue controls.

| Sample GSM No. | Tissue No. | Diagnosis | Age | Sex | Stage | Location |
|---|---|---|---|---|---|---|
| **GSM706244** | FT-103 | nomal skeletal musle | 44 | F | NA | left medial adductor comprtment m |
| **GSM706245** | FT-104 | nomal skeletal musle | 26 | F | NA | tumor, left posterior shoulder |
| **GSM706246** | FT-105 | nomal skeletal musle | 55 | M | NA | tumor, left arm |
| **GSM706247** | FT-262 | adjusant normal skeletal muscle (FT-261, aRMS) | 11 | F | NA | forearm, right |
| **GSM706248** | FT-272 | adjusant normal skeletal muscle (FT-271, eRMS) | 1 | M | NA | kidney, left |
| **GSM706249** | FT-282 | adjusant normal skeletal muscle (FT-281, eRMS) | 6 | M | NA | right testis |
| **GSM706250** | FT-259 | eRMS, unfavourable histology | 8 | M | Grade 2B | testicular, right |
| **GSM706251** | FT-263 | eRMS | 7 | M | Stage -2A | kidney, left |
| **GSM706252** | FT-268 | eRMS | 3 | F | Stage -2A | abdomen |
| **GSM706253** | FT-269 | eRMS | 6 | M | Stage -1 | bladder |
| **GSM706254** | FT-270 | eRMS | 6 | M | NA | jaw, cavity left |
| **GSM706255** | FT-271 | eRMS with spindle cell features | 1 | M | local | kidney, left |
| **GSM706256** | FT-273 | eRMS | 5 | M | NA | retroperitoneal |
| **GSM706257** | FT-281 | aRMS with diffuse anaplasia | 6 | M | Stage -1 Local | right testis |
| **GSM706258** | FT-260 | aRMS | 2 | M | Stage -1 | cervical, posterior |
| **GSM706259** | FT-261 | aRMS | 11 | F | Stage -2 | forearm, right |
| **GSM706260** | FT-266 | aRMS | 1 | F | Stage -1 | neck |
| **GSM706261** | FT-267 | aRMS | 14 | F | NA | breast, right |
| **GSM706262** | FT-276 | aRMS with focal anaplasia | 7 | M | NA | internal anal sphincter |
| **GSM706263** | FT-278 | aRMS | 2 | F | NA | pelvis |
| **GSM706264** | FT-284 | aRMS with diffuse anaplasia | 2 | M | Stage -3 Distant | abdominal pelvic extending into right groin |

| | | | | | | |
|---|---|---|---|---|---|---|
| **GSM706265** | FT-285 | aRMS | 1 | M | NA | left anterior chest wall |
| **GSM706266** | FT-286 | aRMS | 11 | M | NA | left forearm |

Source: adapted from Li et al. (2012).
