# Supplementary material for: Reannotation of Public Transcriptomic Data Identifies Candidate lncRNAs and Putative Regulatory Networks in Rhabdomyosarcoma
Source: Biomedicines. 2026 Jul 22;14(7):1648. doi: 10.3390/biomedicines14071648 (PMC13406521; doi:10.3390/biomedicines14071648)
Supplement: Supplementary file 1 [file biomedicines-14-01648-s001.zip › Supplementary Material S3.pdf]

Supplementary Material S3.

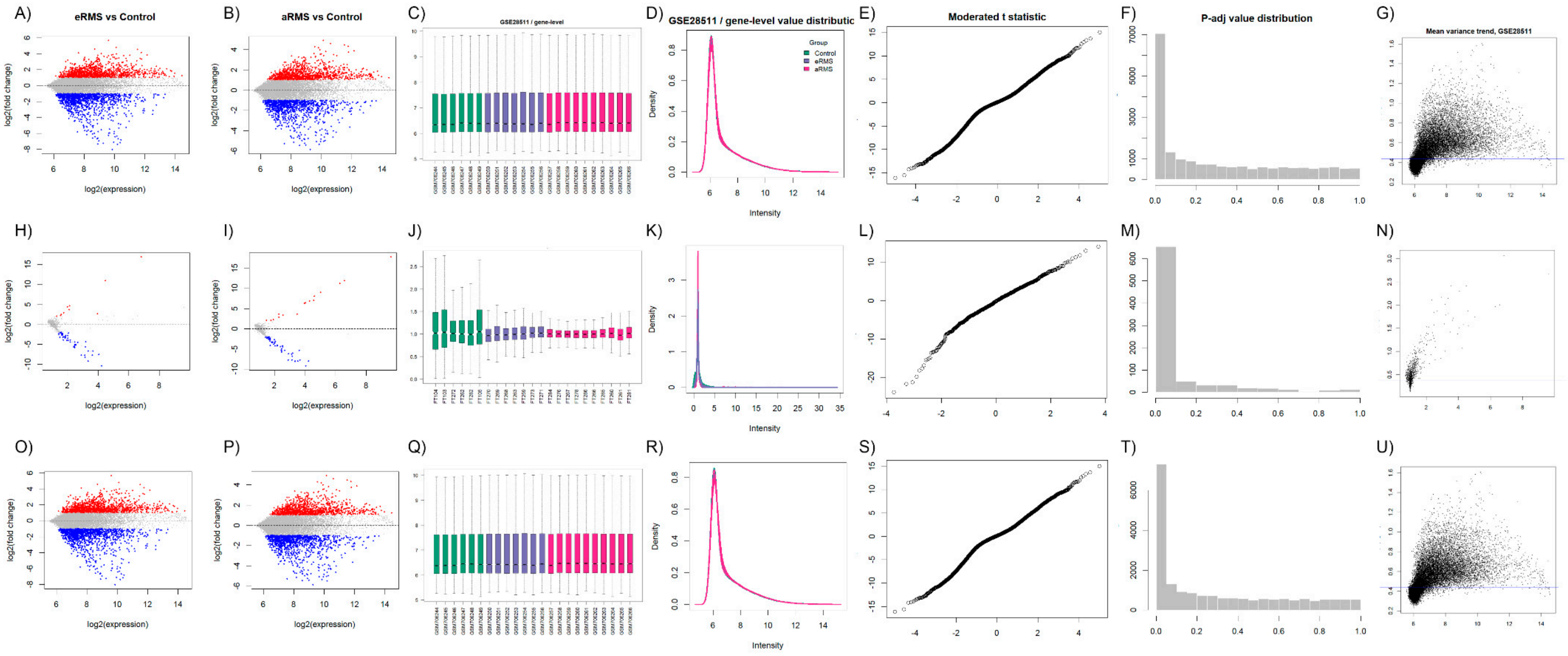

Figure S1 - Differential expression analysis and quality assessment. Plots for lncRNAs before probe reannotation (A-G), miRNAs (H-N), and lncRNAs after probe reannotation (O-U). MD plots show $\log_2$ fold change vs average $\log_2$ expression for eRMS vs Control (A, H, O) and aRMS vs Control (B, I, P); upregulated genes are red, downregulated blue (adjusted $p < 0.05$). Boxplots (C, J, Q) and expression density plots (D, K, R) display sample distributions and normalization. Moderated t-statistic Q-Q plots (E, L, S) assess test quality. Adjusted p-value histograms (F, M, T) show p-value distributions across genes. Mean-variance trend plots (G, N, U) depict the relationship between gene expression mean and variance, evaluating the potential need for precision weighting.
